# Assessment of antibacterial effect of garlic in patients infected with *Helicobacter pylori* using urease breath test

**Published:** 2016

**Authors:** Mahmoud Zardast, Kokab Namakin, Jamil Esmaelian Kaho, Sarira Sadat Hashemi

**Affiliations:** 1 *Birjand Atherosclerosis AndCoronary Artery Research Center, Department of pathology, Birjand University of Medical Sciences (BUMS), Birjand, Iran*; 2 *Department of pediatrics, Birjand University of Medical Sciences (BUMS), Birjand, Iran*; 3 *Birjand University of Medical Sciences, Birjand, Iran (BUMS)*

**Keywords:** *Helicobacter pylori*, *Urease Breath Test (UBT)*, *Garlic*, *Stomach*

## Abstract

**Objective:**

*Helicobacter pylori *(*H. pylori*) is the most common pathogenic bacteria in the stomach. The aim of the current study was to explore the effect of oral garlic administration on bacterial urease activity inside the stomach and its contribution to the treatment of *H. pylori* infection.

**Materials and Methods:**

In this clinical trial, 15 patients were studied quantitatively with Urease Breath Test (UBT). The patients with gastrointestinal symptoms and a positive serum *H. pylori* IgG were enrolled. UBT was performed for each patient in three sessions as follows: at the beginning of the study, an initial UBT was performed based on which, the positive cases entered the study and the negative ones were excluded. Second UBT was done three days later in patients who were not receiving any treatment and were considered as the control, whereas the third UBT was performed three days after prescribing two medium-sized cloves of garlic (3 g) with their meal, twice a day (at noon and in the evening). The collected data were analyzed using ANOVA and Bonferroni tests and the significance level was set at p<0.05.

**Results:**

the mean UBT significantly differed before and after treatment with garlic cloves, being significantly lower after garlic consumption. No meaningful difference was observed in the mean UBT without garlic consumption between the first and second steps.

**Conclusion:**

Raw garlic has anti-bacterial effects against *H. pylori* residing in the stomach and may be prescribed along with routine drugs for the treatment of gastric *H. pylori* infection.

## Introduction

*Helicobacter pylori *(*H. pylori*) is the most common bacterial infection in the world and is the most prevalent gastric bacterial pathogen in humans (Hekmatdoosta et al., 2015 ▶). In developed nations, the prevalence of this bacterium is 20-40% in adults while in developing countries the occurrence rate of this infection is relatively high; in fact, at the age of 20, 70-90% of the population are infected (Fennerty, 2005 ▶; Suerbaum and Michetti, 2002 ▶). *H. pylori* can cause chronic and active gastritis mainly affecting the antrum or fundus; it colonizes in the gastric mucosa and causes gastric inflammation in that region without even directly attacking the tissue. It is also capable of remaining in the area as colonized for a long time without any specific symptoms (Jabbari Nooghabi and jabbariNooghabi, 2008 ▶). *H. pylori* infection is associated with gastrointestinal diseases such as gastritis, peptic and duodenum ulcers, gastric adenocarcinoma, Malt lymphoma and non-gastrointestinal diseases including cardiovascular,thyroidand skin diseases, and other disorders such as autoimmune disorders, anemia, Guillain-Barré syndrome and migraine (Peterson et al., 2000 ▶).

Various treatment regimens which have their own benefits and side effects have been used to eliminate *H. pylori* infection. The most common regimen is triple therapy. This treatment consists of a proton pump inhibitor and two antibiotics, amoxicillin and clarithromycin for 7-14 days (Fuccio et al., 2008 ▶). There is also a quadruple regimen including omeprazole, bismuth and two antibiotics (metronidazole and tetracycline) prescribed for two weeks (Malfertheiner et al., 2000 ▶). 

 The biggest challenge regarding the eradication of *H. pylori* infection is the patients’ low tolerance for medical treatment and the resistance of this organism to antibiotics and other interventions. Eradication by triple therapy in the early studies has shown resistance in over 50% of patients for certain strains (Fauci et al., 2008 ▶), but in recent years, the three-drug regimens have shown progressive reduction in efficacy (less than 80% eradication rate). Not only the quadruple regimen is very costly but also the antibiotics used may cause an undesirable taste in the mouth along with diarrhea and itching. Moreover, among patients who have used metronidazole for a long time, seizure and poly-neuropathy have been reported. *H. pylori* can easily become resistant to clarithromycin and metronidazole; therefore, they cannot be prescribed after a single-treatment course (Meyer et al., 2002 ▶). In addition, Ozturk stated that following resistance to metronidazole, the efficiency of the prescribed treatment would be reduced down to 50% (Ozturk, 2008 ▶). 

Despite different drug therapies, the rate of treatment failure due to resistance is around 5- 20%. Even in many cases with a completed treatment course, recurrence of the disease has been reported (Shoeibi et al., 2010 ▶). In this respect, the use of herbs and herbal medications may be beneficial due to theirnumerous medicinal effects. Todate, the antimicrobial effects of many herbal species (e.g.garlic) on *H. pylori* infection have been studied(Lee et al., 2003 ▶). Garlic has antibiotic, anti-cancer, antioxidant and anti-inflammatory properties; it can lower blood sugar levels and hasprotective effects on the cardiovascular system (Arreola et al., 2015 ▶; Hosseini and Hosseinzadeh, 2015 ▶; Mahdavi-Roshan et al., 2013 ▶; Rios et al., 2015 ▶; Wang et al., 2015 ▶). Furthermore, it is cheaper than many chemical drugs and easily accessible; therefore, it is generally better accepted by the patients resulting in a higher compliance rate (Bokaeian and Bameri, 2013 ▶). Several studies have assessed and reported the antibacterial effects of garlic on different bacteria (Sivam, 2001 ▶; Hosseini-Jazani et al., 2007 ▶). However, the results of McNulty et al. study did not show a significant relationship between garlic consumption and eradication of *H. pylori* infection (McNulty et al., 2008 ▶). 

Given the high prevalence of gastrointestinal diseases in Iran, particularly gastric adenocarcinoma and its association with *H. pylori*, and considering the resistance of *H. pylori* infection to various treatment regimens, finding an appropriate medication for its eradication is of crucial importance. In the current study we aimed to explore the effect of garlic on *H. pylori* infection by using quantitative carbon 14 urease breath test (UBT).

## Materials and Methods

***H. pylori*** **detection techniques**

*H. pylori* can be detected by several methods. Among them, few methods are able to quantitatively evaluate the activity of intra-mucosal live bacteria. Anti-*H.pylori* antibodies do not immediately decrease following treatment initiation and require eat least a few months. Therefore, the immediate level of antibodies in serum does not show the success of treatment. Endoscopy and biopsy are relatively aggressive techniques which cannot assess the early effects of treatment easily. Fecal antigens also show a lot of diversity. Quantitative UBT, on the other hand, can assess the early bacteriostatic effects of bacteria. By this method, the urease activity level of bacteria can be measured in a short time period (Count Per Minutes (CPM)).

**Study design**

In this pre-post-clinical trial, 15 patients with *H. pylori* infection were studied. The patients were enrolled by simple sampling. All patients who referred to the Gastroenterology clinic of Vali-Asr Hospital, Birjand, Iran from May 2013 to Jan 2014 with complaints of dyspepsia and gastrointestinal problems such as abdominal pain, loss of appetite, nausea and flatulence with a positive *H. pylori* IgG test were asked to participate in the study after fully explaining the study protocol. A questionnaire consisting of demographic data was completed for each participant and they were subsequently referred to Shafa Laboratory of Immunology, Birjand, Iran in order to completecarbon-14 urease breath test (UBT) for the diagnosis of active *H. pylori* infection.

The study protocol was approved by the Ethics Committee of Birjand University of Medical Sciences, Birjand, Iran and an informed consent was obtained from each participant prior to study entrance.

**UBT conditions and performance**

The conditions required for performing a quantitative UBT were as follows: fasting for at least 6 hours, no antibiotics and bismuth consumption during the last4 weeks, not having used any proton pump inhibitors, antacids, and histamine receptor blockers (e.g. ranitidine, famotidine and cimetidine) at least for a week and not smoking since one hour before the test. Each patient swallowed a C*14 urea capsule with a glass of water in the seated position and after 10 minutes they blew into a special breathing card; the respiratory card was placed in a plastic bag with aluminum coverage and was counted by a Gamma counter in the least possible time frame. In case of positivity of the gamma counter, the patient was diagnosed with active *H. pylori* infection and entered the study. 

For three days, the patients were banned from consuming garlic or any other preparations of the alliums family, antibiotics and acid-reducing drugs. At the end of the three treatment-free days, the quantitative UBT count was performed once again and recorded as the control. Two medium cloves of raw garlic (3 g) were then administered toeach patient twice a day for 3 days with their daily meals. At the end of the 3-day treatment course, quantitative UBT counting was repeated for the patients, this time regarded as the cases.

**Statistical analysis**

Gamma counter results, expressed as Count Per Minute (CPM), were analyzed by SPSS statistical software ver. 16 and the Kolmogorov-Smirnov test was applied to assess the normal distribution of the collected data. Since the data were normally distributed, intra- group ANOVA and Bonferroni tests were used to analyze the data at the significance level of p<0.05.

## Results

**Figure 1:**
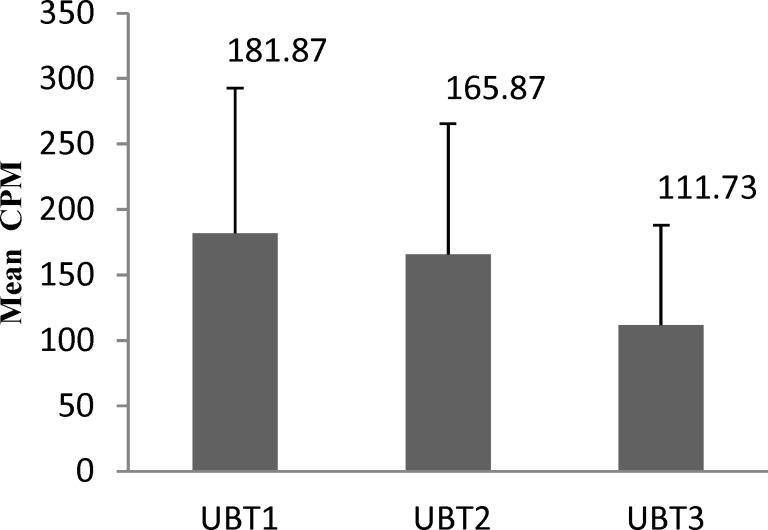
Comparison of the mean CPM of C14-UBT before and after the intervention (CPM: Count per Minute

Fifteen patients, 4 (26.66%) male and 11 (73.33%) female were included in our study. The results of the intra-group analysis of variance showed that the mean UBT was significantly different between the cases (difference between the 2nd and 3rd measurements) and the control (difference between the 1st and 2nd measurements). Bonferroni test showed that the mean CPM in UBT after garlic consumption was significantly lower than the second test (p <0.005) and no significant difference was found between the mean CPM in UBT between the 1st and 2nd measurements (control) (p= 0.169) (Figure 1).

## Discussion

The results of this study showed that consumption of fresh garlic inhibits *H. pylori* activity inside the stomach mucosa to a great extent which indicates a bacteriostatic effect for garlic; bactericidal effects may take place at higher concentrations of garlic or following longer treatment courses. Various studies support the inhibitory effect of garlic on the growth of *H. pylori* (Jonkers et al., 1999 ▶). 

In this context, Cutler and Wilson (2004) ▶ showed that garlic has a wide range of antibacterial activities and is also effective against *H. pylori* infection. Also, Ghobeh et al. (2010) ▶ reported that consumption of 4 gr of garlic powder leads to eradication of bacteria in 87% of *H. pylori* positive individuals. However, McNulty et al. (2008) ▶ study did not reveal any association between consumption of garlic powder and eradication of *H. pylori* infection. 

Numerous studies have investigated the effect of garlic on various bacteria. Tsao et al. (2001) ▶ studied the effect of garlic oil and diallyltrisulfide and diallyltetrasulfide on *Pseudomonas aeruginosa* and showed that these elements potentially prevent or treat nosocomial infections and infections due to antibiotic-resistant strains of bacteria. Kazemizadeh et al. (2011) ▶ studied the effect of garlic extract on *Enterococcus faecalis* whereas Hosseini Jazini et al. (2007) ▶ studied the effect of garlic extract on multidrug-resistant Acinetobacter strains. These studies have pointed out the broad range of antibacterial effects of garlic.

*In vitro* administration of garlic extract at room temperature prevented the activity of gram negative bacteria such as Salmonella and *E. coli* and gram-positive bacteria including streptococcus type A and anthracis type B. Such inhibitory effects of garlic are even more marked than those of penicillin as 1mg of allicin is as effective as 15 standard units of penicillin (Farkhondeh and Aliporyegane., 2012). 

The main components of garlicareorganosulfur (aleinandallicin), organic acids, carbohydrates and vitamins and the most important property of garlic, which is its antimicrobial effect, is attributed to allicin or garlic oil with 162.3 KD molecular weight.

Allicin is not naturally present in garlic cloves, but it is produced after hydrolysis and oxidation of alein. The mechanism(s) for the antimicrobial activity of allicin and garlic extract have not been yet fully investigated; however, a number of mechanisms have been suggested in this respect. Interference with the function of enzymes and proteins containing the sulfhydryl group (SH) is one of such mechanisms. Allicin irreversibly inhibits the SH proteases and NADP-dependent alcohol dehydrogenase (Shapoury et al., 2004 ▶). According to Inder and Qiutang (2002) ▶ study, garlic blocks the activity of nuclear factor kappa-light-chain-enhancer of activated B cells (NF-κB ).This factor increases the expression of inflammatory cytokines and is one of the key molecules in inflammation and cancer. Activation of this nuclear factor is mediated byTLR4 receptor (Toll-like receptors). TLR4 are involved in induction of immune responses. Many of these receptors contain cysteine in their extracellular and cytoplasmic sectors (Iwalokun et al., 2004 ▶). 

In addition, allicin in garlic contains compounds called thiosulfinate that can interact with cysteine. As a result, allicin can react with the cysteine which is in the structure of these receptors resulting in the inhibition of signaling pathways associated with TLR4 on the surface of cellular receptors. Allicin prevents the activation of NF-κB by inhibiting TLR4 signaling. This inhibition is considered as one of the main mechanisms via which garlic induced itsanti-inflammatory effects (Ghobeh et al., 2010 ▶).

Furthermore, *H. pylori* produces Heat Shock Proteins (HSP), urease and lipopolysaccharidase which are absorbed by the stomach epithelial cells. Following absorption, they pass through the mucous and synthesize inflammatory factors such as CRP, IL-8 and TNF-a (Hekmatdoosta et al., 2015 ▶). 

Iwalokun et al. (2004) ▶ findings suggest that garlic extract is effective on the pathogenesis of toxic bacteria by preventing the toxin production. Moreover, it has been noted that allicin can reduce *H. pylori* infection by blocking nitrous synthesis and scavenging bnitrates and free radicals from the body. 

This study had certain limitations; the study population was small and the effect of garlic was not compared with the routine anti-*H. pylori* regimens.

## Note

*Please cite this paper as: *

Zardast M, Namakin K, Esmaelian KahoJ, Hashemi SS. Assessment of Antibacterial antibacterial effect of garlic in patients infected with Helicobacter pylori: assessment of garlic’s effect using Urease Breath Test.Avicenna J Phytomed, 2016; 6 (5): 495-501.
